# Genetic and Phenotypic Landscape of *PRPH2*-Associated Retinal Dystrophy in Japan

**DOI:** 10.3390/genes12111817

**Published:** 2021-11-18

**Authors:** Akio Oishi, Kaoru Fujinami, Go Mawatari, Nobuhisa Naoi, Yasuhiro Ikeda, Shinji Ueno, Kazuki Kuniyoshi, Takaaki Hayashi, Hiroyuki Kondo, Atsushi Mizota, Kei Shinoda, Sentaro Kusuhara, Makoto Nakamura, Takeshi Iwata, Akitaka Tsujikawa, Kazushige Tsunoda

**Affiliations:** 1 Department of Ophthalmology and Visual Sciences, Kyoto University Graduate School of Medicine, Kyoto 606-8507, Japan; tujikawa@kuhp.kyoto-u.ac.jp; 2 Department of Ophthalmology and Visual Sciences, Nagasaki University, Nagasaki 852-8102, Japan; 3 Laboratory of Visual Physiology, Division of Vision Research, National Institute of Sensory Organs, National Hospital Organization Tokyo Medical Center, Tokyo 152-8902, Japan; k.fujinami@ucl.ac.uk; 4 Institute of Ophthalmology, University College London, London EC1V 9EL, UK; 5 Department of Inherited Eye Disease, Moorfields Eye Hospital, London EC1V 2PD, UK; 6 Department of Ophthalmology, Faculty of Medicine, University of Miyazaki, Miyazaki 889-1692, Japan; go_mawatari@med.miyazaki-u.ac.jp (G.M.); nobunaoi@gmail.com (N.N.); ymocl@med.miyazaki-u.ac.jp (Y.I.); 7 Department of Ophthalmology, Nagoya University Graduate School of Medicine, Nagoya 466-8550, Japan; ueno@med.nagoya-u.ac.jp; 8 Department of Ophthalmology, Kindai University, Osaka 589-8511, Japan; kazuki@med.kindai.ac.jp; 9 Department of Ophthalmology, The Jikei University School of Medicine, Tokyo 105-8461, Japan; taka@jikei.ac.jp; 10 Department of Ophthalmology, University of Occupational and Environmental Health, Kitakyushu 807-8555, Japan; kondohi@med.uoeh-u.ac.jp; 11 Department of Ophthalmology, Teikyo University, Tokyo 173-8605, Japan; mizota-a@med.teikyo-u.ac.jp (A.M.); shinodak@med.teikyo-u.ac.jp (K.S.); 12 Department of Ophthalmology, Saitama Medical University, Moroyama 350-0495, Japan; 13 Department of Surgery, Division of Ophthalmology, Kobe University Graduate School of Medicine, Kobe 650-0017, Japan; kusu@med.kobe-u.ac.jp (S.K.); manakamu@med.kobe-u.ac.jp (M.N.); 14 Division of Molecular and Cellular Biology, National Institute of Sensory Organs, National Hospital Organization Tokyo Medical Center, Tokyo 152-8902, Japan; takeshi.iwata@kankakuki.go.jp; 15 Division of Vision Research, National Institute of Sensory Organs, National Hospital Organization Tokyo Medical Center, Tokyo 152-8902, Japan; tsunodakazushige@kankakuki.go.jp

**Keywords:** retinitis pigmentosa, cone-rod dystrophy, macular dystrophy, *peripherin 2* (*PRPH2*), *retinal degeneration slow* (*RDS*)

## Abstract

*Peripherin-2* (*PRPH2*) is one of the causative genes of inherited retinal dystrophy. While the gene is relatively common in Caucasians, reports from Asian ethnicities are limited. In the present study, we report 40 Japanese patients from 30 families with *PRPH2*-associated retinal dystrophy. We identified 17 distinct pathogenic or likely pathogenic variants using next-generation sequencing. Variants p.R142W and p.V200E were relatively common in the cohort. The age of onset was generally in the 40’s; however, some patients had earlier onset (age: 5 years). Visual acuity of the patients ranged from hand motion to 1.5 (Snellen equivalent 20/13). The patients showed variable phenotypes such as retinitis pigmentosa, cone-rod dystrophy, and macular dystrophy. Additionally, intrafamilial phenotypic variability was observed. Choroidal neovascularization was observed in three eyes of two patients with retinitis pigmentosa. The results demonstrate the genotypic and phenotypic variations of the disease in the Asian cohort.

## 1. Introduction

Inherited retinal dystrophy (IRD) refers to a group of diseases characterized by progressive retinal cell death, particularly photoreceptor cell death, caused by genetic mutations. More than 300 causative genes have been reported to date, with a considerable overlap [1]. Recently, gene therapy has become available to patients with pathogenic variants of a specific gene, and other trials are ongoing [2]. Thus, identifying causative genes is becoming increasingly important.

Peripherin-2 (*PRPH2*; online Mendelian inheritance in man ID: 179605, https://www.omim.org/ accessed on 17 November 2021) is one of the causative genes of IRD. The gene is located on chromosome 6p21.2 and contains three exons. The gene was also called *retinal degeneration slow* (*RDS*) because the ortholog was found in a classic animal model, rds mice [3]. *PRPH2* encodes PRPH2 protein, which consists of 346 amino acids and is essential for the proper outer segment formation and maintenance of outer segment disc alignment, both in rod and cone photoreceptors [4]. The gene is generally associated with an autosomal-dominant inheritance pattern, but autosomal recessive [5] and digenic patterns in conjunction with *retinal outer segment membrane protein 1* (*ROM1*) has also been reported [6,7]. Pathogenic variants of *PRPH2* may cause diverse phenotypes such as retinitis pigmentosa (RP), retinitis punctata albescens, cone/cone-rod dystrophy (CRD), and macular dystrophies (MD) [3,8,9,10]. Variable phenotypes were observed in a single family sharing the same variant [11,12,13]. The presence of *ROM1* variants may modify these phenotypic appearances [14] or increase the severity of the disease [15].

Pathogenic variants of *PRPH2* are one of the major causes of IRD. It has been reported that 5.2% of patients with IRD are associated with *PRPH2* in the United Kingdom [13] and 3.9% of patients with RP are associated with *PRPH2* in Spain [16]. The prevalence is particularly high in patients with autosomal dominant CRD or MD; 12% in CRD/MD [17], 19.5% in autosomal dominant CRD/MD [18], and 10.3% in autosomal dominant RP were associated with *PRPH2* [19], respectively.

Meanwhile, the prevalence of *PRPH2* as a causative gene of IRD in Asian populations is relatively low. The prevalence of *PRPH2* as a causative gene of RP is 0.06% in China [20], 1.6% in Korea [21], and 0–3.4% in Japan [22,23,24]. Moreover, the prevalence of *PRPH2* as a causative gene of CRD or MD is 2.3–6.1% [25,26]. Thus, little is known about the genetic and phenotypic spectrum of *PRPH2*-associated IRD in Asia.

In this multicenter joint study, we recruited patients with *PRPH2*-associated IRD from all over Japan and reported their phenotypic and genotypic characteristics.

## 2. Materials and Methods

This study adhered to the tenets of the Declaration of Helsinki and was approved by the Ethics Committees of the participating institutions in Japan (National Institute of Sensory Organs, National Hospital Organization Tokyo Medical Center [Reference: R18-029] and Kyoto University Graduate School of Medicine [Reference: G0746]). All patients who participated in the study provided written informed consent.

### 2.1. Clinical Examinations

All patients underwent comprehensive ophthalmological examination, including best-corrected visual acuity (BCVA) measurement, slit-lamp ophthalmoscopy, fundus photography, fundus autofluorescence imaging, optical coherence tomography (OCT), visual field test, electroretinogram (ERG), and electrooculogram (EOG), if available. ERG and EOG were recorded in accordance with the standards of the International Society for Clinical Electrophysiology of Vision [27,28,29]. Clinical diagnosis was made at each institution and reviewed by the consortium. In the present study, phenotype subgroups were defined based on clinical manifestations reported in a previous study. RP was defined as a progressive retinal dystrophy initially often presenting peripheral atrophy with generalized rod dysfunction greater than cone dysfunction. CRD was defined as a progressive retinal dystrophy initially often presenting macular atrophy with generalized cone dysfunction greater than rod dysfunction. MD was defined as a progressive retinal dystrophy presenting macular atrophy with confined macular dysfunction despite no abnormalities of generalized cone and rod functions [30].

**Figure 1:**
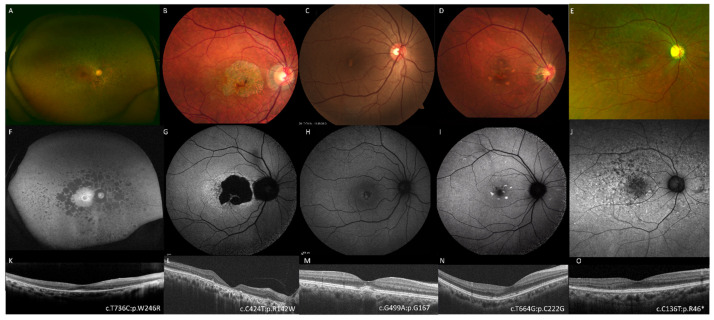
Phenotypic spectrum of patients with *PRPH2*-associated retinal dystrophy. Images of the fundus (**A**–**E**), fundus autofluorescence (**F**–**J**), and optical coherence tomography (**K**–**O**). Some cases showed peripheral atrophy compatible with retinitis pigmentosa (left column, patient KYT6553), macular atrophy compatible with cone/cone-rod dystrophy (the second column, patient NISO 1014-001), Best disease-like foveal deposit (the third column, patient JKI167-1314), pattern dystrophy-like flecks (fourth column, patient NISO112-112), and Stargardt disease-like multiple flecks (the right column, patient UOEH188-1). Causative variants and protein changes are shown.

In addition to the phenotype subgroups, we investigated the presence of clinical factors such as macular atrophy, peripheral atrophy, Best disease-like deposits, and multiple flecks on retinal imaging because some patients showed overlapping phenotypes and clinical diagnosis may obscure the characteristics (Figure 1).

We obtained family history and assumed the mode of inheritance as autosomal dominant if two generations or more were affected; autosomal recessive if there was parental consanguinity or siblings from normal parents were affected; X-linked if the diseased occurred in multiple generations but without male-to-male transmission and only males were affected. 

### 2.2. Genetic Screening

While this study focused on *PRPH2*, the screening was conducted as a part of comprehensive genetic screening of patients with IRD. Genomic DNA was extracted from peripheral blood samples and analyzed using next-generation sequencer as previously reported. [22,30,31,32,33]. Briefly, we employed targeted exome sequencing for case series of 2014 [22], whole genome sequencing for Kyoto University cases after 2014 [32], and whole exome sequencing for the other participants. Subsequently, target analysis of retinal disease-associated genes was performed. We analyzed all the variants in exons and their boundaries (±2 bp) that were detected on the genes registered in Retinal Information Network (RetNet; Available online: https://sph.uth.edu/retnet/ (accessed on 1 September 2021)). The identified variants were filtered based on the allele frequency in the Human Genetic Variation database (HGVD, a database of allele frequency in the general Japanese cohort; Available online: http://www.hgvd.genome.med.kyoto-u.ac.jp/ (accessed on 1 September 2021), Genome Aggregation Database (gnomAD; Available online: https://gnomad.broadinstitute.org/), and 1000 Genomes (Available online: http://www.internationalgenome.org/ (accessed on 1 September 2021)). Missense variants were evaluated using seven in silico prediction programs: MutationTaster (http://www.mutationtaster.org/; accessed on 1 September 2021), FATHMM (http://fathmm.biocompute.org.uk/9; accessed on 1 September 2021), Combined Annotation Dependent Depletion (https://cadd.gs.washington.edu/; accessed on 1 September 2021), SIFT (https://www.sift.co.uk/; accessed on 1 September 2021), PROVEAN (http://provean.jcvi.org/index.php; accessed on 1 September 2021), and Polyphen 2 (http://genetics.bwh.harvard.edu/pph2/; accessed on 1 September 2021). Splice site alteration was assessed using Human Splicing Finder (http://umd.be/Redirect.html; accessed on 1 September 2021). The evolutionary conservation score for each variant was calculated using PhyloP30way (https://ccg.epfl.ch/mga/mm9/phylop/phylop.html; accessed on 1 September 2021), and PhastCons30way (https://bioconductor.org/packages/release/data/annotation/html/phastCons30way.UCSC.hg38.html; accessed on 1 September 2021). Variant classification was performed according to the American College of Medical Genetics and Genomics (ACMG) guidelines [34]. Candidate variants were confirmed by Sanger sequencing in the index patient and their family members, if possible.

### 2.3. Statistical Analysis

Decimal visual acuity was converted to logarithm of the minimum angle of resolution (logMAR) for statistical analysis. Counting finger and hand motion were regarded as logMAR 2.0 and 2.3, respectively [35]. Comparisons between the two groups were performed using the Mann–Whitney U test or chi-square test, as appropriate. Associations between the clinical factors were assessed using the Spearman’s rank correlation test. All statistical analyses were performed using IBM SPSS Statistics 26 (IBM Japan, Tokyo, Japan).

## 3. Results

A total of 40 patients from 30 families with 17 distinct *PRPH2* variants were identified. Details of the patients are summarized in Table 1. Some cases have been reported previously [36,37]. Twenty patients were men and 20 were women. Based on comprehensive examinations, patients were phenotypically classified into the RP (*n* = 16), CRD (*n* = 7), and MD (*n* = 17) subgroups. Among patients with MD, four had Best disease-like deposits, and seven had Stargardt disease or pattern dystrophy-like multiple flecks. Three of the four patients with Best disease-like deposits had subnormal EOG. Seven of 16 patients with RP had macular atrophy in addition to typical peripheral atrophy. Meanwhile, two patients with CRD and one patient with MD had peripheral atrophy. Common primary complaints were reduced visual acuity or central visual field loss (16 patients, 40%), night blindness (12 patients, 30%), photophobia (4 patients, 10%), and peripheral visual field loss (1 patient, 2.5%). Three (7.5%) patients had no symptoms at the time of diagnosis.

**Figure 2:**
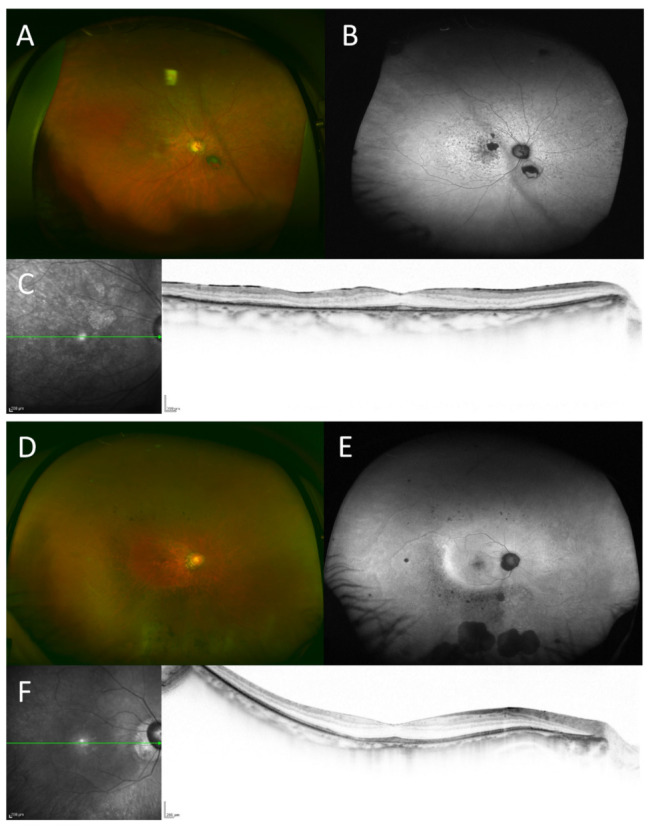
Illustrative cases (patient KYT6144, A-C and KYT6274, D-F) with different phenotypes in a family sharing the same *PRPH2* variant (c.499G>A). Images of the fundus (**A**) and fundus autofluorescence (**B**) in the 88-year-old mother revealing macular atrophy. Thinning of outer retinal layer and disruption of ellipsoid zone are observed in the parafoveal area. (**C**) Electroretinogram of the patient showing normal rod and cone response. Meanwhile, midperipheral atrophy, predominantly in the inferior retina, is observed in her 61-year-old daughter (**D**,**E**). The macula is relatively spared; however, thinning of the outer retinal layer toward the periphery is observed. (**F**) Electroretinogram of the patient showing non-recordable rod and barely recordable cone responses.

Most patients (from 16 families) had autosomal dominant inheritance of *PRPH2*-associated retinal dystrophy, whereas 11 patients had sporadic disease. We could not determine the inheritance pattern in one patient. The pedigree charts of two patients were not available. Some discrepancies in the phenotype subgroups within families were noted. Illustrative cases are presented in Figure 2. An 88-year-old woman had pattern-dystrophy-like MD and her 61-year-old daughter had RP. The mother showed a lower limit of normal range but still recordable rod and cone responses in ERG, the daughter showed a non-recordable rod and barely recordable cone responses.

**Figure 3:**
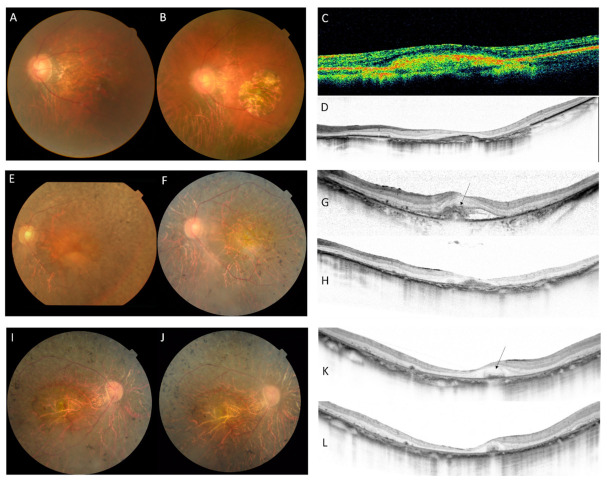
Illustrative cases with pathogenic variants in *PRPH2* (patient KYT6274 and KYT6074) who developed choroidal neovascularization (CNV) in association with retinitis pigmentosa. Patient KYT6274 had high myopia and developed CNV at the age of 49 years. (**A**) Macular atrophy progressed in 13 years. (**B**) CNV at 2 years after development (**C**) CNV at 13 years after development (**D**) Unfortunately, OCT images of new-onset CNV were not available. Patient KYT6074 developed the CNV in the left and right eyes at the age of 55 and 65 years, respectively. (**E**,**G**,**I**,**K**) The patient received multiple injections of anti-vascular endothelial growth factor agents. Fibrotic atrophy and macular thinning were observed at the final visit (**F**,**H**,**J**,**L**). CNVs are indicated with arrows on OCT images.

Two patients developed choroidal neovascularization (CNV). Both patients were classified into the RP phenotype subgroup. The images of the fundus and OCT are presented in Figure 3. One of the patients had high myopia with a spherical equivalent of −10.5 diopter. Anti-vascular endothelial growth factor therapy was not available at that time. Visual acuity declined from 0.7 (20/30) to 0.1 (20/200) in 15 years with the progression of macular atrophy. Another patient presented with bilateral CNV without evident drusen. She had received >60 intravitreal injections of anti-vascular endothelial growth factor agents for the left eye in 13 years and part of the treatment course was reported elsewhere [37]. She developed CNV in the right eye 8 years after the start of treatment in the left eye. Visual acuity declined from 0.7 (20/30) to 0.2 (20/100) in the left and from 1.0 (20/20) to 0.4 (20/50) in 13 and 5 years, respectively.

The age of the patients ranged from 28 to 88 years. The age of onset was generally in the 40′s; however, some patients had the onset as early as 5 years of age. No association was found between the age of onset and sex or visual acuity; however, patients with RP tended to develop symptoms earlier than patients with CRD or MD (31.2 vs. 40.9 years, *p* = 0.161).

Visual acuity of the patients ranged from hand motion (logMAR equivalent 2.3) to 1.5 (Snellen equivalent 20/13, logMAR equivalent −0.18). No significant difference in BCVA was noted among patients with RP, those with CRD, and those with MD. As expected from the irreversible and progressive nature of the disease, BCVA tended to be worse in elderly patients (Spearman’s correlation = 0.36, *p* = 0.22).

**Figure 4:**
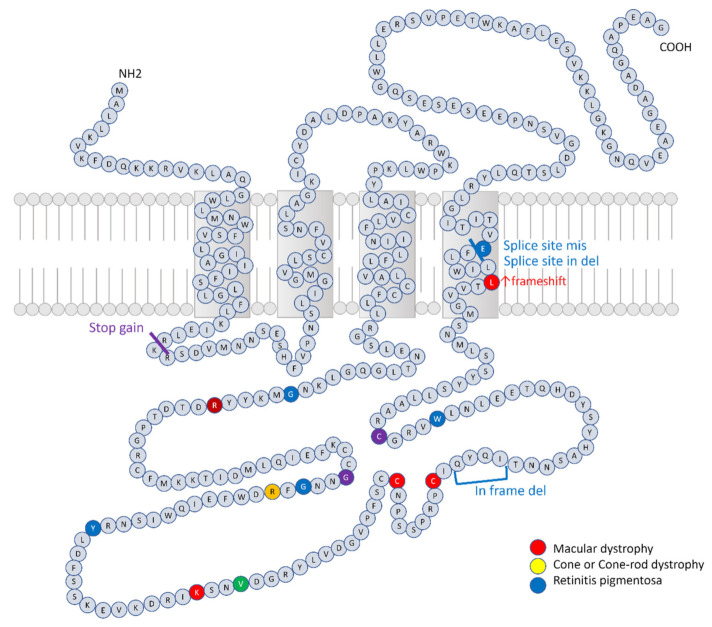
Schematic model of amino acid sequence of peripherin and variants found in the present study. (The presentation style has been adapted from Boon et al. Prog Ret Eye Res 2008 [3]. Sequence is based on NP_000313.2). Missense or in-frame deletions are shown as colored residues and premature termination and frameshift variants are shown as colored bars. When a locus is associated with various phenotype, they were indicated by mixed colors e.g., red + yellow = orange, yellow + blue = green, red + blue = purple, and red + yellow + blue = brown.

The data of the detected variants are presented in Table 2 and Table 3. Eleven variants were previously reported, whereas six were novel. Twelve variants were missense, two were splice site, one was a frameshift, one was a stop gain, and one was an in-frame deletion. The locations of the variants in the amino acid sequence are illustrated in Figure 4. All missense variants were located in the D2 loop of the protein. None of the cases had likely pathogenic or pathogenic variants in *ROM1*.

## 4. Discussion

Here, we present the clinical and genetic features of 40 patients with *PRPH2*-associated retinal dystrophy. To the best of our knowledge, this is the largest cohort among Asian ethnicities. This study confirmed phenotypic variability, even within the same family. The present data provide novel insights into genotype-phenotype associations in East Asian ethnicity.

Previous reports have suggested association between the location of the variant in *PRPH2* and clinical phenotype. Specifically, variants are mostly found in the D2 loop [13,43,46,47,48], which is critical for protein–protein interactions. Variants that cause autosomal dominant RP tend to accumulate between Lys193 and Glu226 [3]. Particularly, missense mutations in Pro210 to Pro216 cause autosomal dominant RP [3]. Patients diagnosed with CRD, RP, and STGD tend to have variants in exon 1, whereas patients with Best disease and pattern dystrophy tend to have variants in exon 2 [13,43]. Patients with p.Arg172Trp present earlier onset than those with other alleles [43]. Some of these findings are compatible with the findings of the present cohort; 14 of 17 (82.3%) identified variants were in the D2 loop. Six of 16 patients (from two of ten families) with RP had variants between Lys193 and Glu226. The Best disease- or pattern dystrophy-like deposits were found in 10 patients. Five of these 10 patients had variants in exon 2. However, the age of onset in four patients with p.Arg172Trp was 45–60 years. The results reveal the difficulty in establishing a clear genotype-phenotype correlation.

The prevalence of founder variants was found to be different. In Caucasians, c.424C>T (p.Arg142Trp) and c.514C>T (p.Arg172Trp) are common causative variants. Other recurrent variants are c.136C>T (p.Arg46*), c.422A>G (p.Tyr141Cys), c.441del (p.Gly148Alafs*5), c.514C>G (p.Arg172Gln), c.554T>C (p.Leu185Pro), c.584G>T (p.Arg195Leu), c.623G>A (p.Gly208Asp), c.629C>G (p.Pro210Arg), c.646C>T (p.Pro216Ser), c.647C>T (p.Pro216Leu), c.715C>T (p.Gln239*), c.866C>T (p.Ser289Leu), and c.828+3A>T [13,43,46,49]. Among these variants, c.514C>T was reported in a previous study in Japan [50] and was found in four patients from four families in our cohort. The variant c.424C>T was found in four families and c.136C>T was found in two families. However, other variants were not found in the present study or in previous studies in Japan [42,44,50,51,52,53,54,55,56,57,58]. Instead, c.599T>A (p.V200E) reported in a previous study in Japan [51] was found in seven patients from three families in the present study. Of note, the locations of the institutions where the previous study was conducted and where the patients were recruited in the present study were 1600 km apart. The results indicate ethnicity-based characteristic variants in the Japanese population, as well as shared variants among multiple ethnicities. The inter-ethnic differences should be considered when interpreting *PRPH2* variants in patients with IRD.

CNV was relatively common (2/40, 5%) in our cohort. CNV occasionally occurs in IRD, but is not a common complication, especially in RP [59]. One of the patients was considered to have myopic CNV. The other patient might have been complicated with neovascular age-related macular degeneration (AMD), but drusen, a hallmark of AMD, was not evident. We assumed that the patient developed dystrophy-associated CNV. The development of CNV is generally discussed in association with pattern dystrophy [3,60]; however, it can be seen in the RP phenotype as previously reported [61]. Considering that anti-vascular endothelial growth factor therapy is an effective treatment for CNV. Patients should be advised to visit the eye clinic when they notice acute vision loss and/or metamorphopsia.

This study has some limitations. First, the prevalence of *PRPH2*-associated dystrophy could not be determined. Each institution recruited patients independently and the criteria to proceed to genetic testing may have been different in each institution. Patients with a dominant inheritance pattern may be more willing to undergo genetic testing. Nevertheless, 40 cases were sufficient to determine the phenotypic variability within the cohort. Second, the pathogenicity of each variant is based on the standard criteria but not on solid biological evidence. Although we systematically applied ACMG guidelines and graded all identified variants as pathogenic or likely pathogenic, there is still a chance that some of these variants are bystanders and pathogenic variants are present in other loci or genes. Finally, we did not intensively investigate the disease modifying effect of *ROM1* variants. While none of the patients had likely pathogenic or pathogenic variants in *ROM1*, variants filtered out or beyond the target lesion may modify the phenotype.

## 5. Conclusions

We analyzed 40 Japanese patients with *PRPH2*-associated retinal dystrophy and confirmed the genotypic and phenotypic variations of the disease in the Japanese population. Further studies involving multiple ethnicities would enhance our understanding of the disease.

## Figures and Tables

**Table 1:** Clinical characteristics of patients with *PRPH2*-associated retinal dystrophy.

ID	Family ID	Inheritance Trait	Sex	Age	Onset Age	Visual Acuity * Right/Left	Macular Atrophy	Peripheral Atrophy	Best Disease-Like Deposit	Flecks	Phenotype SubGroup	Variants
NISO 1034-001	1	AD	F	57	10	0.05/0.00	−	+	−	−	RP	c.136C>T (p.R46 *)
UOEH 188-2	2	AD	F	42	Unknown	0.00/−0.08	+	−	−	+	MD	c.136C>T (p.R46 *)
UOEH 188-1	2	AD	F	67	Unknown	0.00/0.00	+	−	−	+	MD	c.136C>T (p.R46 *)
KYT 6074	3	AD	F	68	43	0.00/0.15	+	+	−	−	RP	c.410G>A (p.G137D)
KYT 6322	4	AD	M	53	46	0.15/0.10	−	+	−	−	RP	c.410G>A (p.G137D)
MYZ 098-001	5	AD	F	42	No symptom	0.05/0.05	+	−	−	−	CRD	c.424C>T (p.R142W)
MYZ 098-002	5	AD	M	69	58	2.30/2.30	+	+	−	−	RD	c.424C>T (p.R142W)
KYT 6703	6	AD	M	73	Unknown	1.10/1.10	+	−	−	−	MD	c.424C>T (p.R142W)
KYT 4296	6	AD	M	78	70	1.30/0.22	+	−	−	−	MD	c.424C>T (p.R142W)
KND 129-75	7	Sporadic	F	56	Unknown	0.15/0.15	+	−	−	−	MD	c.424C>T (p.R142W)
NISO 1014-001	8	Sporadic	M	64	58	0.15/0.00	+	−	−	−	MD	c.424C>T (p.R142W)
KYT 6274	9	AD	F	61	41	0.00/0.70	−	+	−	−	RP	c.499G>A (p.G167S)
KYT 6144	9	AD	F	88	Unknown	0.00/0.05	+	−	−	+	MD	c.499G>A (p.G167S)
JKI 167-1314	10	Sporadic	F	50	Unknown	−0.08/0.00	−	−	+	−	MD	c.499G>A (p.G167S)
MYZ 040-001	11	Sporadic	F	75	60	0.52/0.22	−	+	−	−	RP	c.508G>A, (p.G170S)
NGY 0255	12	n.a.	M	43	Unknown	0.52/0.40	+	−	−	−	CRD	c.514C>T, (p.R172W)
NISO 1010-001	13	Sporadic	M	52	45	0.22/0.05	+	−	−	+	MD	c.514C>T, (p.R172W)
NGY 0196	14	Sporadic	M	62	60	0.40/0.22	+	−	−	−	MD	c.514C>T, (p.R172W)
JKI 275-1927	15	Sporadic	M	57	56	0.30/0.52	+	−	−	−	MD	c.514C>T, (p.R172W)
KB 001-01	16	AD	M	66	46	0.22/0.70	+	;	−	−	RP	c.551A>C (p.Y184S)
KYT 6399	17	AD	F	73	25	1.70/1.70	+	−	−	−	MD	c.589A>G (p.K197E)
MYZ 093-001	18	AD	M	61	23	2.30/2.30	+	+	−	−	RP	c.599T>A (p.V200E)
MYZ 093-002	18	AD	M	64	45	1.52/2.00	+	+	−	−	RP	c.599T>A (p.V200E)
MYZ 093-004	18	AD	M	33	20	0.00/0.00	+	−	−	−	RP	c.599T>A (p.V200E)
MYZ 093-005	18	AD	M	31	28	−0.08/−0.08	+	−	−	−	CRD	c.599T>A (p.V200E)
MYZ 101-001	19	AD	F	32	29	0.30/0.30	+	−	−	−	CRD	c.599T>A (p.V200E)
MYZ 101-003	19	AD	F	52	5	1.30/1.22	+	−	−	−	CRD	c.599T>A (p.V200E)
MYZ 066-001	20	AD	F	40	32	0.40/0.52	+	+	−	−	CRD	c.599T>A (p.V200E)
NGY 0148	21	AD	M	49	Unknown	0.00/0.00	+	−	−	+	MD	c.641G>A (p.C214Y)
NISO 112-112	22	Sporadic	M	49	14	−0.08/0.00	+	+	−	−	MD	c.664T>G (p.C222G)
KND 109-38	23	AD	F	28	8	0.00/0.00	−	+	−	−	RP	c.670_681del (p.Q224_I227del)
KND 109-51	23	AD	M	30	14	−0.08/−0.08	−	+	−	−	RP	c.670_681del (p.Q224_I227del)
KND 109-52	23	AD	M	67	27	2.00/1.52	+	+	−	−	RP	c.670_681del (p.Q224_I227del)
KYT 6553	24	AD	F	58	15	0.05/0.05	−	+	−	−	RP	c.736T>C (p.W246R)
JKI 060-0665	25	AD	M	48	45	−0.08/−0.18	−	−	+	−	RP	c.748T>G (p.C250G)
KYT 6237	26	n.a.	F	49	Unknown	−0.18/0.05	−	−	+	−	MD	c.748T>G (p.C250G)
KYT 6102	27	Sporadic	F	53	37	0.52/-0.08	+	−	−	+	MD	c.748T>G (p.C250G)
JKI 077-0735	28	Sporadic	M	58	56	−0.08/0.00	+	−	+	+	MD	c.809_810del (p.L270Pfs *30)
NISO 4002-001	29	Sporadic	F	38	6	1.70/1.52	−	+	−	−	RP	c.828G>T (p.E276D)
NISO 3001-001	30	AD/AR	F	59	50	0.52/0.52	+	+	−	−	RP	c.828_828+5 delGGTAGGinsC

* Visual acuity is presented as the logarithm of minimum angle of resolution. Abbreviations: AD, autosomal dominant; AR, autosomal recessive; RP, retinitis pigmentosa; MD, macular dystrophy (including Stargardt disease, pattern dystrophy, and Best disease phenotypes); CRD, cone dystrophy, cone-rod dystrophy; n.a., not available.

**Table 2:** Allele frequency of the *PRPH2* variants identified in the present study.

Varaiant ID	Family ID	Nucleotide Change, Amino Acid Change	Position (hg19)	Coding Impact	dbSNP ID	HGVD	iJGVD 3.5k	1000 Genome	GnomAD Allele Frequency								
									Allele Frequency					Coverage in GnomAD Exomes Samples			
									East Asian	South Asian	African	European (Non-Finnish)	Total	Mean Coverage	Median Coverage	% of Samples Over 20× Coverage	Reference
1	1,2	c.136C>T, (p.Arg46Ter)	Chr6:42689937	Nonsense	rs61755771	0.0000%	0.0000%	NA	NA	NA	0.0062%	0.0009%	0.0035%	95.9	100	99.88%	Meins (1993) Hum Mol Genet. [38]
2	3, 4	*c.410G>A, (p.Gly137Asp)*	Chr6:42689663	Missense	rs527236097	0.0000%	0.0000%	NA	NA	NA	NA	NA	NA	87.2	100	99.90%	Novel (listed on ClinVar, SCV001240217.1)
3	5, 6, 7, 8	c.424C>T, (p.Arg142Trp)	Chr6:42689649	Missense	rs61755783	0.0000%	0.0000%	NA	NA	NA	NA	0.0009%	0.0016%	89.3	100	99.30%	Hoyng (1996) Am J Ophthalmol [39]
4	9, 10	c.499G>A, (p.Gly167Ser)	Chr6:42689574	Missense	rs527236098	0.0000%	0.0000%	NA	NA	NA	NA	NA	NA	98.9	100	100.00%	Testa (2005) Br J Ophthalmol [40]
5	11	c.508G>A, (p.Gly170Ser)	Chr6:42689565	Missense	rs61755791	0.0000%	0.0000%	NA	0.0163%	0.0033%	NA	NA	0.0016%	99.2	100	99.99%	Kohl (1998) Acta Anat (Basel) [41]
6	12, 13, 14, 15	c.514C>T, (p.Arg172Trp)	Chr6:42689559	Missense	rs61755792	0.0000%	0.0000%	NA	NA	NA	NA	NA	NA	99.2	100	99.97%	Wells (1993) Nat Genet [9]
7	16	c.551A>C, (p.Tyr184Ser)	Chr6:42689522	Missense	rs62645926	0.0000%	0.0000%	NA	NA	NA	NA	NA	NA	99.1	100	99.89%	Nakazawa (1996) Arch Ophthalmol [42]
8	17	c.589A>G, (p.Lys197Glu)	Chr6:42672342	Missense	rs62645931	0.0000%	0.0000%	NA	NA	NA	NA	NA	NA	89.0	100	99.88%	Reeves (2020) Hum Mutat [43]
9	18, 19, 20	c.599T>A, (p.Val200Glu)	Chr6:42672332	Missense	rs62645932	0.0000%	0.0000%	NA	NA	NA	NA	NA	NA	89.4	100	99.89%	Nakazawa (1996) Retina [44]
10	21	c.641G>A, (p.Cys214Tyr)	Chr6:42672290	Missense	rs61755804	0.0000%	0.0000%	NA	NA	NA	NA	NA	NA	87.1	100	99.96%	Trujillo (2000) Hum Mutat [45]
11	22	*c.664T>G, (p.Cys222Gly)*	Chr6:42672267	Missense	NA	0.0000%	0.0000%	NA	NA	NA	NA	NA	NA	82.0	100	99.87%	Novel
12	23	*c.670_681del, (p.Gln224_Ile227del)*	Chr6:42672250	In frame	NA	0.0000%	0.0000%	NA	NA	NA	NA	NA	NA	78.8 81.3	100 100	99.59% 99.85%	Novel
13	24	*c.736T>C, (p.Trp246Arg)*	Chr6:42672195	Missense	rs61755817	0.0000%	0.0000%	NA	NA	NA	NA	NA	NA	69.2	100	89.02%	Novel
14	25, 26, 27	c.748T>G, (p.Cys250Gly)	Chr6:42672183	Missense	NA	0.0000%	0.0000%	NA	NA	NA	NA	NA	NA	67.6	100	82.58%	Katagiri (2018) Ophthalmic Genet [36]
16	28	c.809_810delT, (p.Leu270ProfsTer30)	Chr6:42672121	Frameshift	NA	0.0000%	0.0000%	NA	NA	NA	NA	NA	NA	64.4 64.4	100 100	68.64% 68.72%	Peeters (2021) Hum Mutat [46]
17	29	*c.828G>T, (p.Glu276Asp)*	Chr6:42672103	Missense (splice site alteration)	NA	0.0000%	0.0000%	NA	NA	NA	NA	NA	NA	63.6	100	65.95%	Novel
18	30	*c.828_828+5delGGTAGGinsC*	Chr6:42672098	Splice site alteration	NA	0.0000%	0.0000%	NA	NA	NA	NA	NA	NA	63.3 63.6	100 100	65.21% 65.95%	Novel

Abbreviation: NA, not available. Reference: NM_000322.5, ENST00000230381.5, GRCh37.p13. Novel variants are presented in italic.

**Table 3:** Results of *in-silico* prediction and ACMG classification.

Variant ID	Family ID	Nucleotide Change, Amino Acid Change	General Prediction						Functional Prediction								Conservation				ACMG Classification							
			MutationTaster			FATHMM			SIFT			PROVEAN			Polyphen2		PhyloP30way		PhastCons30way		Verdict	Classification Categories						
			Prediction	Accuruacy	Converted Rankscore	Prediction	Score	Converted Rankscore	Prediction	Score	Converted Rankscore	Prediction	Score	Converted Rankscore	Prediction	Score	Mammalian	Mammalian Rankscore	Mammalian	Mammalian Rankscore								
1	1, 2	c.136C>T, p.(Arg46*)	Disease causing automatic	1	0.81	NA	NA	NA	NA	NA	NA	NA	NA	NA	NA	NA	1.1759	0.7892	0.986	0.5005	Pathogenic	PVS1 (strong)	PM2	PP1	PP3	PP5		
2	3, 4	*c.410G>A, (p.Gly137Asp)*	Disease causing	0.9997	0.49	Damaging	−2.2	0.8689	Damaging	0.003	0.6824	Damaging	−2.83	0.5971	Probably Damaging	0.963	1.026	0.4595	0.9629	0.4369	Likely Pathogenic	PM1	PM2	PP1	PP3	PP5		
3	5, 6, 7, 8	c.424C>T, p.(Arg142Trp)	Disease causing automatic	0.9304	0.37	Tolerated	−1.28	0.7938	Damaging	0.000	0.9125	Damaging	−4.08	0.7474	Probably Damaging	0.998	0.2119	0.2406	0.9969	0.6203	Pathogenic	PM1	PM2	PM5	PP1	PP3	PP5	
4	9, 10	c.499G>A, p.(Gly167Ser)	Disease causing	1	0.81	Damaging	−5.54	0.9918	Damaging	0.000	0.9125	Damaging	−5.29	0.8432	Probably Damaging	1.000	1.026	0.4595	0.762	0.3244	Pathogenic	PS1	PM1	PM2	PM5	PP1	PP3	PP5
5	11	c.508G>A, p.(Gly170Ser)	Disease causing	0.9998	0.49	Damaging	−2.24	0.8719	Tolerated	0.100	0.3052	Neutral	−1.13	0.2911	Possibly Damaging	0.822	1.026	0.4595	0.949	0.4173	Likely Pathogenic	PM1	PM2	PP3	PP5			
6	12, 13, 14, 15	c.514C>T, p.(Arg172Trp)	Disease causing	0.962%	0.38	Tolerated	−1.36	0.8012	Damaging	0.000	0.9125	Damaging	−5.33	0.8460	Probably Damaging	0.999	1.1759	0.7892	0.9919	0.5410	Pathogenic	PM1	PM2	PM5	PP3	PP5	\	
7	16	c.551A>C, p.(Tyr184Ser)	Disease causing	1	0.81	Tolerated	−1.13	0.7772	Damaging	0.000	0.9125	Damaging	−6.16	0.9021	Probably Damaging	1.000	1.138	0.6469	1	0.8628	Likely Pathogenic	PM1	PM2	PP2	PP5			
8	17	c.589A>G, (p.Lys197Glu)	Disease causing	0.9953	0.43	Tolerated	4.1	0.0294	Damaging	0.035	0.4371	NA	NA	NA	Probably Damaging	0.759	1.138	0.6469	1	0.8628	Likely Pathogenic	PM1	PM2	PP3	PP5			
9	18, 19, 20	c.599T>A, p.(Val200Glu)	Disease causing	1	0.81	Tolerated	3.88	0.0357	Damaging	0.000	0.9125	Damaging	−3.85	0.7235	Possibly Damaging	0.941	1.312	0.9471	1	0.8628	Likely Pathogenic	PM1	PM2	PP1	PP3	PP5		
10	21	c.641G>A, p.(Cys214Tyr)	Disease causing	1	0.81	Damaging	−3.25	0.9353	Damaging	0.000	0.9125	Damaging	−10.22	0.9893	Probably Damaging	1.000	1.026	0.4595	0.999	0.7043	Pathogenic	PM1	PM2	PM5	PP3	PP5		
11	22	*c.664T>G, p.(Cys222Gly)*	Disease causing	1	0.81	Damaging	−2.08	0.8601	Damaging	0.000	0.9125	Damaging	−11.10	0.9934	Probably Damaging	0.995	1.312	0.9471	0.9959	0.5952	Likely Pathogenic	PM1	PM2	PM5	PP3			
12	23	*c.670_681del, p.(Gln224_Ile227del)*	NA	NA	NA	NA	NA	NA	NA	NA	NA	NA	NA	NA	NA	NA	NA	NA	NA	NA	Likely Pathogenic	PM1	PM2	PM4	PP1	PP3		
13	24	*c.736T>C, (p.Trp246Arg)*	Disease causing	1	0.81	Tolerated	−1.26	0.7918	Damaging	0.003	0.6824	Damaging	−7.52	0.951	Probably Damaging	0.996	1.312	0.9471	0.995	0.5772	Likely Pathogenic	PM1	PM2	PM5	PP3			
14	25, 26, 27	c.748T>G, p.(Cys250Gly)	Disease causing	1	0.81	Damaging	−7.44	0.9988	Damaging	0.000	0.9125	Damaging	−10.84	0.9922	Probably Damaging	1.000	1.312	0.9471	0.995	0.5772	Likely Pathogenic	PM1	PM2	PM5	PP3	PP5		
16	28	c.809_810delTC, p.(Leu270Profs*30)	NA	NA	NA	NA	NA	NA	NA	NA	NA	NA	NA	NA	NA	NA	1.307	0.8849	0.039	0.1619	Likely Pathogenic	PVS1 (moderate)	PM2	PP3	PP5			
17	29	*c.828G>T, p.(Glu276Asp)*	Disease causing	0.9999	0.53	Tolerated	−1.3	0.7957	Damaging	0.023	0.4819	Neutral	−2.17	0.4902	Possibly Damaging	0.680	1.0219	0.3987	0.8949	0.3737	Likely Pathogenic	PVS1 (strong)	PM2					
18	30	*c.828_828+5delGGTAGGinsC*	NA	NA	NA	NA	NA	NA	NA	NA	NA	NA	NA	NA	NA	NA	NA	NA	NA	NA	Likely Pathogenic	PVS1 (strong)	PM2					

Abbreviations: ACMG, American College of Medical Genetics and Genomics; NA, not available. Reference: NM_000322.5, ENST00000230381.5, GRCh37.p13. Novel variants are presented in italic. PVS, pathogenicity very strong; PS, pathogenicity strong; PM, pathogenicity moderate; PP, pathogenicity supporting.

## Data Availability

Data are available upon reasonable request.
